# Molecular Recognition of the Hybrid-Type G-Quadruplexes in Human Telomeres

**DOI:** 10.3390/molecules24081578

**Published:** 2019-04-22

**Authors:** Guanhui Wu, Luying Chen, Wenting Liu, Danzhou Yang

**Affiliations:** 1 Department of Medicinal Chemistry and Molecular Pharmacology, College of Pharmacy, Purdue University, 575 W Stadium Ave, West Lafayette, IN 47907, USA; wu1109@purdue.edu (G.W.); chen2321@purdue.edu (L.C.); liuwting@mail2.sysu.edu.cn (W.L.); 2 Purdue Center for Cancer Research, 201 S University St, West Lafayette, IN 47906, USA; 3 Purdue Institute for Drug Discovery, 720 Clinic Dr, West Lafayette, IN 47907, USA

**Keywords:** human telomeres, G-quadruplex, G4, anticancer drug, solution structure, molecular recognition, rational drug design, hybrid-2, hybrid-1, epi-berberine, platinum-tripod

## Abstract

G-quadruplex (G4) DNA secondary structures formed in human telomeres have been shown to inhibit cancer-specific telomerase and alternative lengthening of telomere (ALT) pathways. Thus, human telomeric G-quadruplexes are considered attractive targets for anticancer drugs. Human telomeric G-quadruplexes are structurally polymorphic and predominantly form two hybrid-type G-quadruplexes, namely hybrid-1 and hybrid-2, under physiologically relevant solution conditions. To date, only a handful solution structures are available for drug complexes of human telomeric G-quadruplexes. In this review, we will describe two recent solution structural studies from our labs. We use NMR spectroscopy to elucidate the solution structure of a 1:1 complex between a small molecule epiberberine and the hybrid-2 telomeric G-quadruplex, and the structures of 1:1 and 4:2 complexes between a small molecule Pt-tripod and the hybrid-1 telomeric G-quadruplex. Structural information of small molecule complexes can provide important information for understanding small molecule recognition of human telomeric G-quadruplexes and for structure-based rational drug design targeting human telomeric G-quadruplexes.

## 1. Introduction

**Figure 1:**
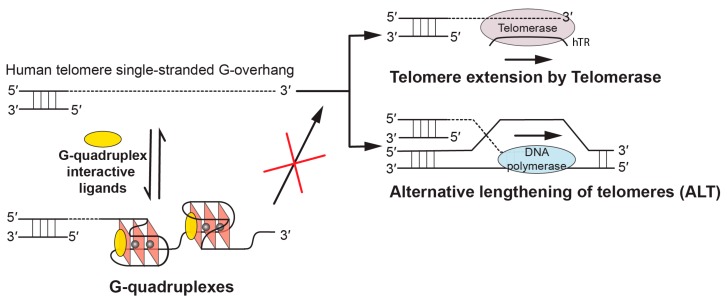
Biological implications of targeting telomeric G-quadruplexes using G-quadruplex-interactive ligands. In human tumors, the free single-stranded telomere G-overhang can be extended by telomerase, which reverse transcribes the template region of its RNA subunit (hTR), or by alternative lengthening of telomeres (ALT), which copies telomeric template DNA via homologous recombination. G-quadruplex-interactive ligands can promote the formation of and stabilize the telomeric G-quadruplex structures, thus inhibiting telomere extension processes which are required for the indefinite growth of human tumors.

Human telomeres are essential DNA-nucleoprotein complexes capping the terminus of chromosomes to protect the chromosomes from end-to-end fusion and degradation [1,2]. Telomeres play an important role in genomic stability [3], aging [4], and cancers [5]. Human telomeric DNA consists of TTAGGG tandem repeats 5–20 kb in length, terminating in a 30–500 nucleotide single-stranded 3′ overhang [6]. In human somatic cells, telomeric DNA undergoes a progressive shortening with every cell division because of the end-replication problem [7]. When the telomere length reaches a critical limit, the cell undergoes apoptosis or senescence. However, as one of the hallmarks of cancer, cancer cells counteract the progressive loss of the telomere length to achieve limitless replication potential [5,8]. One telomere length maintenance mechanism is provided by a reverse transcriptase called telomerase (Figure 1), which is activated in 85–90% of cancer cells [9,10,11]. Another mechanism is the alternative lengthening of telomeres (ALT) pathway (Figure 1), which maintains telomere integrity in 10%–15% of cancer cells that lack detectable telomerase activity [11,12,13,14,15].

**Figure 2:**
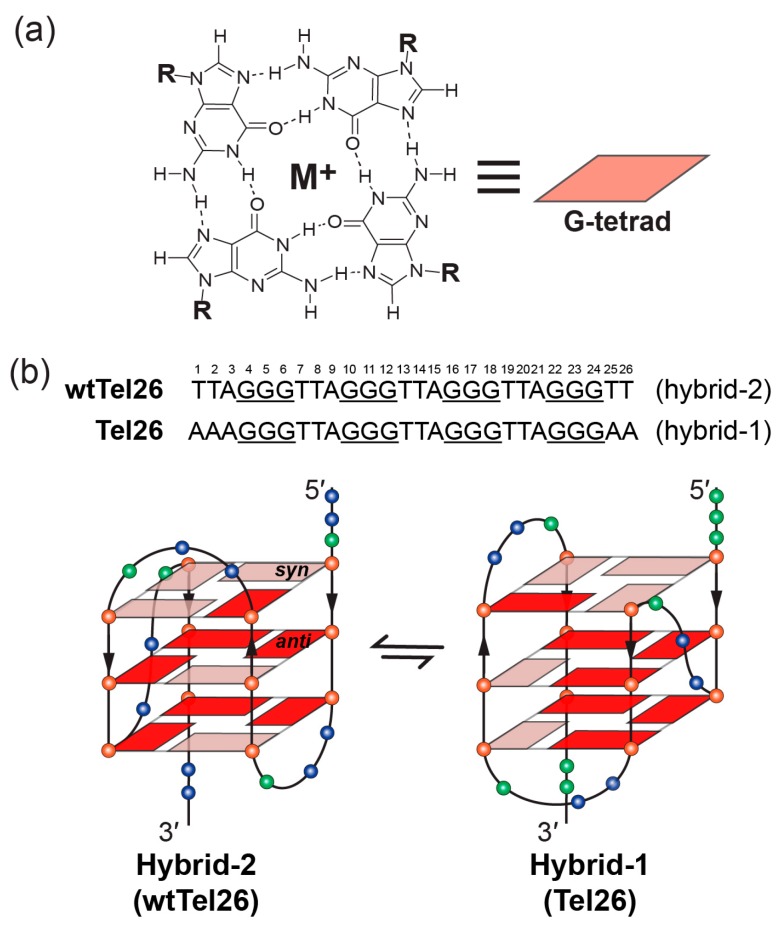
Schematic illustration of G-tetrad and the folding pattern of hybrid-2 and hybrid-1 telomeric G-quadruplexes. (**a**) Schematic representation of G-tetrad. Monovalent cations (M^+^), such as K^+^ or Na^+^, are required for the G-tetrad formation. (**b**) Human telomeric sequences predominantly fold into two hybrid-type G-quadruplexes with an equilibrium between hybrid-1 (PDB ID: 2HY9) and hybrid-2 (PDB ID: 2JPZ) forms. The human telomeric sequences used to determine hybrid-2 (wtTel26) and hybrid-1 (Tel26) structures are shown on the top. Strand polarities are indicated as the black arrows. Glycosidic conformations of guanine nucleotides are marked as follows: *syn*-pink and *anti*-red. Different nucleotides are represented as follows: Thymine, blue sphere; guanine, red sphere; and adenine, green sphere.

G-quadruplexes have been found to form in human telomeres and G-quadruplex formation inhibits the activity of telomerase [16]. G-quadruplex formation is suggested to be related to replication stress caused by oncogenic stimulation of hyperproliferation, or by genome instability, and helicases have been shown to play an important role in resolving G-quadruplex structures and G-quadruplex-induced genome instability [17,18,19]. DNA G-quadruplexes are non-canonical four-stranded helical structures, formed in DNA sequences, that are rich in guanine nucleotides [20]. They are assembled by stacked guanine tetrad planes (G-tetrad, Figure 2a) in which four guanines are held together by Hoogsteen hydrogen bonds. Physiologically relevant monovalent cations, especially K^+^, are required to stabilize G-quadruplex structures by coordinating and forming electrostatic interactions with the eight guanine carbonyl oxygen atoms of the two adjacent G-tetrads [21,22,23]. The therapeutic possibilities of targeting telomeric G-quadruplexes to inhibit telomerase were first reported in 1997 [24] and have been actively pursued [25,26,27,28]. For example, in this special issue [29], multiple reports are on telomeric G-quadruplex-interactive ligands [30,31,32,33,34,35,36,37]. G-quadruplex-interactive ligands have been shown to inhibit telomerase (Figure 1) and induce apoptosis in cancer cells [38]. In addition, G-quadruplex-interactive ligands were also shown to inhibit the alternative lengthening of telomeres (ALT) pathway which maintains telomere stability in a telomerase-independent manner [39,40,41,42,43], and can thus avoid drug resistance of direct telomerase inhibitors through activating the ALT pathway (Figure 1) [44]. Therefore, human telomeric G-quadruplexes are considered as attractive cancer-specific drug targets.

The formation of G-quadruplexes in the single-stranded 3′-overhang at the human telomere end most likely involves intramolecular G-quadruplex structures. However, intramolecular human telomeric G-quadruplexes are structurally polymorphic and may adopt different conformations, including two equilibrating hybrid-type structures [45,46,47,48,49,50,51] in K^+^ solution and in cells [52,53,54]. The structure polymorphism appears to be an intrinsic property of the highly conserved telomeric sequence in higher eukaryotes with a TTA loop sequence [55]. Interconversion between different human telomeric G-quadruplexes appears to be kinetically slow, albeit with small energy differences between different conformations, indicating a high-energy intermediate(s) [45,56,57,58,59]. 

The previous studies showed the human telomeric G-overhang predominantly forms two hybrid-type G-quadruplex structures (Figure 2b) in physiologically relevant K^+^-containing solutions, named hybrid-1 [45,47,49] and hybrid-2 [46,50]. These two structures coexist in K^+^ solution in an equilibrium [55]. Their structures are unique in strand orientation, G-tetrad arrangements, loop arrangements, as well as 5′- and 3′-capping, compared to the parallel structures formed in the crystalline form [60] and those predominantly found in the oncogene promoters [20]. The 26-mer wtTel26 sequence, which was the wild-type four-repeats human telomeric sequence with flanking segments at both ends, adopts hybrid-2 conformation (Figure 2b, left) [46]. The first, third, and fourth G-tracts go in one direction and the second G-tract in the opposite direction. In addition, the first, second, and third G-tracts are connected with two TTA lateral loops, while the third and fourth G-tracts are linked with a TTA side loop, i.e., a lateral-lateral-side loop arrangement. The glycosidic bonds of guanine nucleotides in a G-tetrad can adopt either *syn* or *anti* conformations. In the hybrid-2 structure, the top G-tetrad has reversed glycosidic conformations (*syn:anti:syn:syn*) from those of the bottom two G-tetrads (*anti:syn:anti:anti*) (Figure 2b, left). On the other hand, the 26-mer Tel26 sequence with modified 3′- and 5′-flanking segments adopts hybrid-1 conformation (Figure 2b, right) [47]. The first, second, and fourth G-tracts go in one direction and the third G-tract in the opposite direction, with a side-lateral-lateral loop arrangement. The top G-tetrad has reversed glycosidic conformations (*syn:syn:anti:syn*) relative to the other two G-tetrads (*anti:anti:syn:anti*) (Figure 2b, right).

The two hybrid-type telomeric G-quadruplexes have unique capping structures, determined by the flanking and loop sequences together with the folding topology. In the hybrid-2 structure (Figure 2b, left), the 5′-flanking residues, the second TTA lateral loop, and the A21 residue of the third TTA reversal loop are above the top G-tetrad, but are not well structured. In contrast, a well-defined T:A:T triad is formed at the 3′-end of hybrid-2 telomeric G-quadruplex by the interactions between T8 and A9 of the first TTA lateral loop, as well as the T25 of the 3′-end flanking segment. The 3′-capping structure is specific to the hybrid-2 structure and it is not possible to form in the hybrid-1 structure. Additionally, this capping structure is important for the stability for the hybrid-2 structure, as demonstrated by mutational analysis [46]. In the hybrid-1 structure (Figure 2b, right), both 5′-end and 3′-end capping structures are well-defined. The 5′-end capping structure, an adenine-triple, is formed by the A3 residue of the 5′-flanking residues, the A9 residue of the first TTA strand-reversal side loop, and the A21 residue of the third TTA lateral loop. The 3′ capping structure, A:T base pair, is formed by the A25 of 3′-end flanking segment and the T14 of the second lateral loop form.

**Figure 3:**
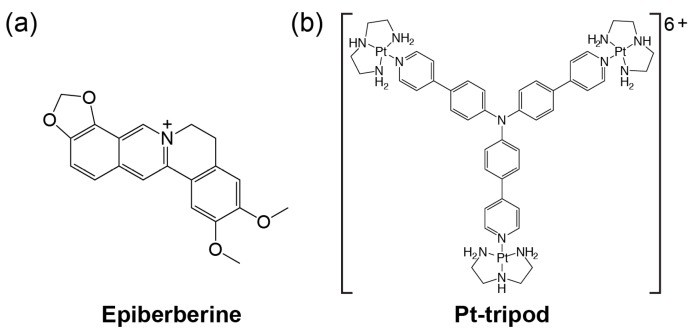
The chemical structures of epiberberine (**a**) and Pt-tripod (**b**).

Structural data of small-molecule complexes of the human telomeric G-quadruplexes can provide important information for understanding specific recognition by small molecules and for structure-based rational drug design targeting human telomeric G-quadruplexes. Only a handful solution structures are available of ligand complexes of human telomeric G-quadruplexes [61,62,63,64]. This review will focus on the two recent solution structural studies from our labs. We use NMR spectroscopy to elucidate the solution structures of a 1:1 complex between a medicinal natural product epiberberine (Figure 3a) and the hybrid-2 telomeric G-quadruplex [64], and 1:1 and 4:2 complexes between a Pt-containing small molecule (Figure 3b) and the hybrid-1 telomeric G-quadruplex [63].

## 2. Molecular Recognition of Human Telomeric Hybrid-2 G-Quadruplex by Epiberberine

**Figure 4:**
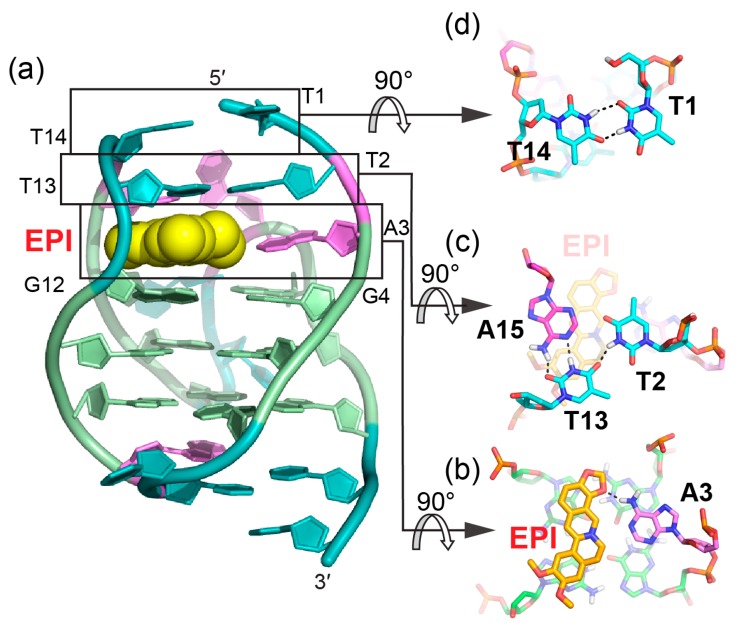
The specific recognition of human hybrid-2 telomeric G-quadruplex by EPI (PDB ID: 6CCW) (**a**) Cartoon representation of 1:1 EPI/hybrid-2 complex. Different nucleotides are marked as follows: Thymine, cyan; adenine, pink; and guanine, green. The EPI molecule is shown as the yellow spheres. (**b**–**d**) Top view of the EPI:A3 quasi-triad plane (**b**), T2:T13:A15 triad plane (**c**), and T1:T14 pair (**d**). Potential hydrogen bonds are shown in black dashed lines.

Protoberberines are a class of isoquinoline alkaloids with antitumor activities [65,66]. Notably, berberine and some of its derivatives have been shown to stabilize telomeric G-quadruplexes and inhibit telomerase activity [67]. Epiberberine (EPI) was found to exhibit great fluorescence enhancement induced by human telomeric sequences in K^+^ solution [68]. Our data show that EPI specifically binds the hybrid-2 human telomeric G-quadruplex structure and we have determined the solution structure of the 1:1 complex of EPI and hybrid-2 human telomeric G-quadruplex by NMR [64]. The NMR solution structure of the complex shows that the EPI molecule (Figure 3a) forms a well-defined 1:1 complex with the hybrid-2 human telomeric G-quadruplex. This complex is stabilized by extensive hydrogen-bonding, base-stacking and electrostatic interactions (Figure 4a). In the free telomeric hybrid-2 structure, the flanking segment and the T13-T14-A15 lateral loop at the 5′ end are disordered. Upon binding, EPI stacks on the 5′-external G-tetrad and extensive rearrangement occurs in the 5′-end flanking residues and lateral loop to form a multi-layer drug-binding pocket. The A3 base from the 5′-flanking strand is recruited by EPI to form a ‘quasi-triad plane’ which is intercalated between the 5′-G-tetrad and two additional capping layers (Figure 4a). Specifically, EPI stacks over the tetrad G12 and G16, with its N7 centered over the 5′-tetrad (Figure 4b), and A3 covers the center of the G4–G22. Importantly, a hydrogen bond is formed between A3/NH6 and the oxygen of the methylenedioxy ring of the EPI, which stabilizes the EPI-A3 plane. The positively charged N7 atom (Figure 3a) of the EPI is in proximity to the O6 atoms of the 5′ external tetrad guanines, likely stabilizing the complex analogous to the K^+^ cations within the central channel of the G-core. Above the EPI:A3 plane, a T:T:A triad is formed from T2 of the 5′ flanking strand and T13 and A15 from the lateral loop. The triad is stabilized by a hydrogen-bonding network, with two hydrogen-bond interactions between T13 and A15 to form a reversed Watson–Crick base pair (Figure 4c) and one hydrogen bond between T2 and T13. In fact, the stable formation of the T:T:A triad results in the appearance of a signature NMR imino proton peak of T13 arisen from the unique triad conformation of the specific EPI-binding pocket within the hybrid-2 telomeric G-quadruplex. Finally, this triad is capped by a hydrogen-bonded T1:T14 base pair, which stacks over the triad and further stabilizes the overall complex (Figure 4d).

**Figure 5:**
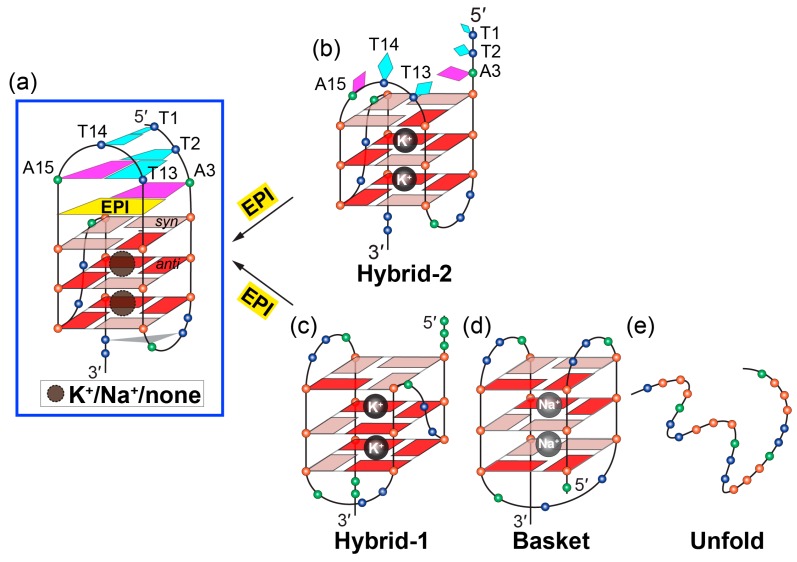
Conversion of human telomeric G-quadruplex structures to the hybrid-2 form induced by EPI. (**a**–**b**) EPI binding induces extensive rearrangement of previously disordered 5′-flanking and lateral loop segments (**b**) to form a well-defined four-layer binding pocket (**a**) specific to hybrid-2 telomeric G-quadruplex. (**c**–**e**) Other human telomeric G-quadruplex forms, including hybrid-1 (**c**) and basket (**d**), or the free telomeric sequence in the absence of salt (**e**) can be converted to the hybrid-2 form as the addition of EPI molecules. The specific recognition of human hybrid-2 telomeric G-quadruplex by EPI (PDB ID: 6CCW).

The optimal recognition of EPI is specific to the hybrid-2 folding topology and the human telomeric DNA loop sequence TTA. In the EPI-hybrid-2 complex, the second TTA loop adopts a lateral loop conformation above the 5′-tetrad, which provides ideal orientation for the pairing interaction with the 5′-flanking segment to form the highly stable capping structures of the T:T:A triad and T:T pair (Figure 5a,b). In the hybrid-1 structure, the third TTA loop at the 5′-end (Figure 5c) is offset by 90° relative to the second lateral loop at the 5′-end of the hybrid-2 structure and, thus, cannot form the optimal stable capping structures for the EPI binding pocket. In the basket-type telomeric G-quadruplex structure, the diagonal loop and the 3′-flanking segments are both at the same end of the 5′-flanking (Figure 5d), sterically hindering the EPI binding. Human telomeric G-quadruplexes bear inherent structure polymorphism, predominantly with two hybrid-type G-quadruplexes in equilibrium between hybrid-2 and hybrid-1 structures under physiologically relevant solution conditions. Remarkably, EPI is able to convert hybrid-1, basket, and unfolded telomeric G-quadruplex structures to the hybrid-2 form, independent of available solution cations (Figure 5). This is the only such compound reported to date. The highly specific and selective binding of EPI to the hybrid-2 structure likely shifts the equilibrium between different forms in solution, resulting in the overall conversion of other telomeric G-quadruplex structures to the hybrid-2 form.

The EPI-hybrid-2 complex structure reveals several features which enable specific recognition of the hybrid-2 human telomeric G-quadruplex. EPI contains a crescent-shaped asymmetric stacking moiety that can only stack over two tetrad guanines, allowing the recruitment of a flanking base partner to co-stack over the 5′-G-tetrad and lock the EPI position. The pairing with the recruited adenine and the central location of positively charged N7 together anchor the position and orientation of EPI above the 5′ external G-tetrad. In addition, the appropriately positioned hydrogen-bond acceptors in the methylenedioxy ring E of EPI enable it to optimally hydrogen-bond with the recruited adenine (Figure 3a and Figure 4b). This is highlighted by the observation that the structurally similar berberine alkaloids berberine, palmatine, and coptisine, which only differ in the positions of the methylenedioxy ring and methoxy groups, are unable to form a well-defined complex with the telomeric G-quadruplex. This molecular-level recognition information can only be obtained from detailed structural study and is important for rational drug design of improved analogs targeting the hybrid-2 human telomeric G-quadruplex.

## 3. Molecular Recognition of Human Telomeric Hybrid-1 G-Quadruplex by Pt-Tripod in Monomeric and Dimeric Complexes

Recently, a series of platinum (II) compounds was found to bind telomeric G-quadruplex and suppress telomerase activity [69,70]. Among them, a Pt-based tripod (Pt-tripod) showed strong in vitro and in vivo anticancer activity upon light irradiation. This tripod is a non-planar compound with a central tertiary amine connecting three arms, each bearing two aromatic rings and a cationic platinum complex with a three-fold symmetry (Figure 3b). Interestingly, Pt-tripod binds to the intramolecular hybrid-1 human telomeric G-quadruplex formed by the Tel26 sequence and forms well-defined monomeric and dimeric Pt-tripod-Tel26 complexes dependent on the drug-DNA ratio [63].

**Figure 6:**
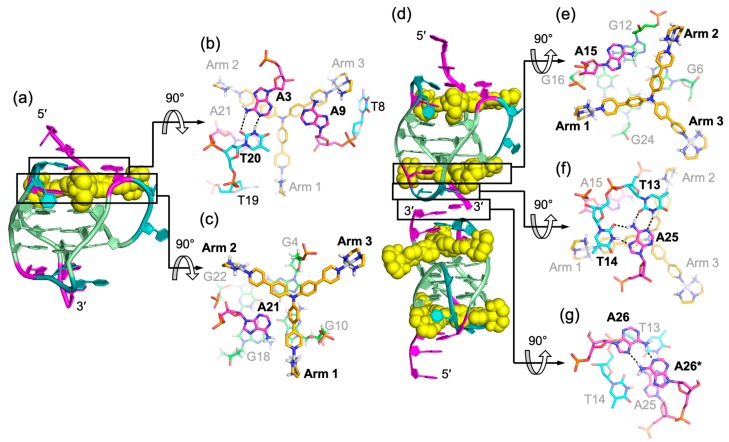
Solution structure of 1:1 and 4:2 Pt-tripod/hybrid-1 G-quadruplex complexes (PDB ID: 5Z80 and 5Z8F). (**a**) Cartoon representation of 1:1 Pt-tripod/hybrid-1 complex. Different nucleotides are marked as follows: Thymine, cyan; adenine, pink; and guanine, green. The Pt-tripod molecule is shown as the yellow spheres. (**b**–**c**) Top views of the A3:A9:T20 triad plane (**b**) and Pt-tripod:A21 plane at 5′ site (**c**). (**d**) Cartoon representation of 4:2 Pt-tripod/hybrid-1 complex. (**e**–**g**) Structural details of the 3′ site binding pocket. Bottom views of the Pt-tripod:A15 plane (**e**), T13:T14:A25 triad plane (**f**), and the A26:A26* (A26 from each hybrid-1) base pair (**g**). Potential hydrogen bonds are shown in black dashed lines.

At the 1:1 ratio, Pt-tripod binds to the 5′-end of the hybrid-1 Tel26 to form a well-defined 1:1 Pt-tripod–Tel26 complex (Figure 6a). In the 1:1 complex, the ligand binding induces a large conformational rearrangement at the 5′ end. Pt-tripod intercalates between A3 of the 5′-flanking segment and G4 of the 5′-tetrad and recruits A21 from the third lateral loop to form a Pt-tripod-A21 paired plane above the 5′-external tetrad (Figure 6c), replacing the adenine triad formed by A3:A9:A21 in the free Tel26 G-quadruplex. The Pt-tripod covers G10 and G4 by its arm 1 and arm 2, respectively, with the central tertiary amine nitrogen right above the G4 and G10 edge, while A21 stacks over the G18 and G22. Above the Pt-tripod-A21 plane, A3 and T20 form a hydrogen-bonded reversed Watson–Crick base pair, while A9 from the first side loop also positions in the same plane to cover the aromatic moiety of arm 3 (Figure 6b).

At the 3:1 Pt-tripod–DNA ratio, Pt-tripod binds the hybrid-1 Tel26 to predominately form a well-defined 4:2 Pt-tripod–Tel26 3′–3′ dimeric complex (Figure 6d) with a twofold symmetry. In the 4:2 Pt-tripod–Tel26 complex, Pt-tripod binds at both the 5′- and 3′-ends of Tel26 G-quadruplex. In addition to the 5′-end complex, the second ligand induces a large conformational rearrangement at the 3′-end to form a well-defined binding pocket, in which Pt-tripod intercalates between A25 and G24 of the 3′-tetrad and recruits A15 from the second lateral loop to form a Pt-tripod-A21 paired plane above the 3′-external tetrad (Figure 6e). Here, arm 1 and arm 2 of the Pt-tripod cover G24 and G6, respectively, with the central tertiary amine nitrogen above the G6 and G24 edge, while A15 stacks over G12 and G16. While the 4:2 dimeric complex is induced by Pt-tripod binding, the binding of Pt-tripod at the 3′-end also appears to be facilitated by the 3′-end interlocking interface in the dimeric complex, including a well-defined hydrogen-bonded T:A:T triad (Figure 6f) and a A:A base pair (Figure 6g). This stable three-layer binding pocket at the 3′ dimeric interface gives rise to a less dynamic binding of Pt-tripod as compared to the 5′-complex. The T14:A25 base pair observed at the 3′-end in the free Tel26 G-quadruplex is completely rearranged, with the glycosidic conformation of T14 changed from *anti* to *syn* and A25 from *syn* to *anti*. It is noted that this unique 3′ dimeric interface appears to be specific to the modified 3′-flanking sequence of Tel26.

**Figure 7:**
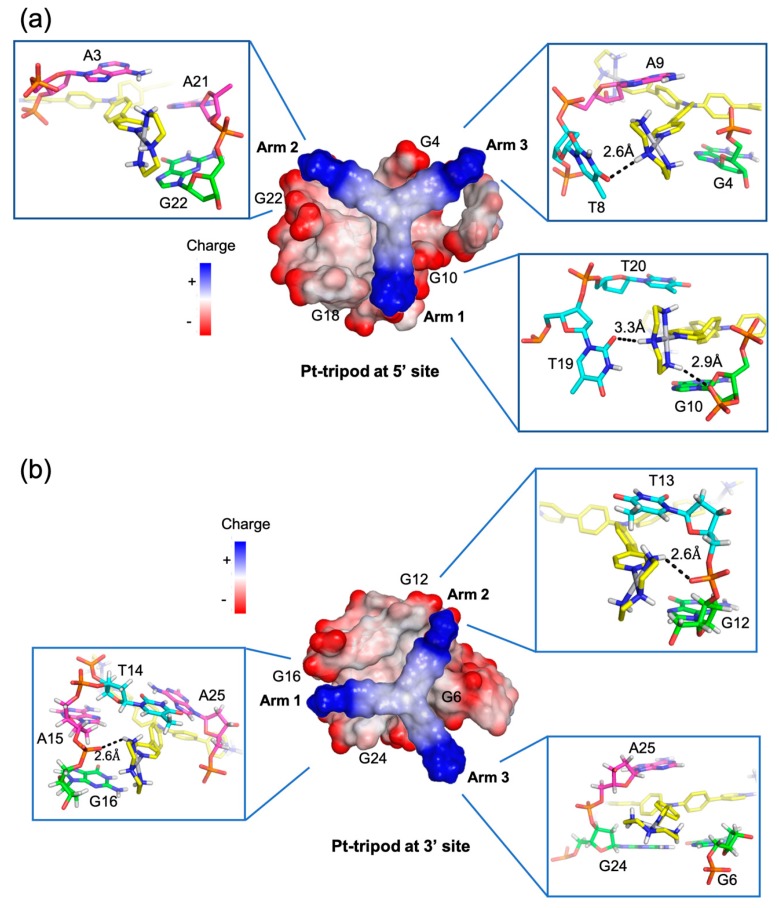
Arm-groove interactions of Pt-tripod at 5′ and 3′ sites in the 4:2 Pt-tripod/hybrid-1 complex. (**a**) Top view of Pt-tripod at the 5′ site of hybrid-1 without capping structure (middle). The binding pocket surface is color coded according to the charge. The interaction details between each arm and groove are shown in enlarged view (corners). (**b**) The same information is shown for Pt-tripod at the 3′ site. Potential hydrogen bonds are shown in black dashed lines.

A unique feature of the two Pt-tripod–Tel26 complexes is the anchoring of the three positively charged arms of Pt-tripod in three G-quadruplex grooves, which defines and locks the position of Pt-tripod in the complexes (Figure 7). Interestingly, the anchoring of the Pt-tripod arms appears to avoid the lateral loops, which may cause potential steric hindrance. Each arm of Pt-tripod stretches into a groove without a lateral loop and thereby positions its outer platinum complexes for interactions with the loop residues and DNA backbone. These include electrostatic interactions of the positively charged platinum moiety of the three arms with the negatively charged phosphate backbone, hydrogen-bonding interactions, and π–π interactions of the three arms with loop and flanking residues. As the tertiary amine nitrogen of Pt-tripod is located above the edge of the external G-tetrad in both complexes, the aromatic moieties of arm 1 and arm 2 of both ligands stack over a guanine of the external G-tetrad, whereas the arm 3 almost completely extends into the groove without much stacking interaction with the external G-tetrads. In the 5′-end complex (Figure 7a), however, the arm 3 of Pt-tripod extensively interacts with the propeller side loop, with A9 stacking over its first aromatic ring and T8 hydrogen-bonded with its terminal moiety. While A21 of the third lateral loop at the 5′-end is recruited to pair with Pt-tripod, T19 of the same loop interacts with arm 1 through hydrogen bonding with T20 further stacking over A21. Arm 2 is intercalated between A3 of the flanking segment and G4 of the 5′-tetrad, with its platinum-moiety interacting with the phosphate backbone in the groove. In the 3′-end complex (Figure 7b), arm 1 and arm 2 of Pt-tripod show electrostatic and potential hydrogen bonding interactions with the phosphate backbone.

## 4. Insights Obtained from the Complex Structures

EPI and Pt-tripod both induce extensive conformational rearrangements of flanking and loop residues after binding to the human telomeric G-quadruplexes. The EPI-hybrid-2 G-quadruplex and 1:1 and 4:2 Pt-tripod-hybrid-1 G-quadruplex complexes all include ligand-induced well-defined multi-layer binding pockets involving the external tetrad, flanking, and loop residues. Such rearrangements are more extensive than most G-quadruplex-interactive-ligand complexes, particularly those observed with the parallel-stranded G-quadruplexes that lack the lateral loops. Therefore, the importance of interactions with flanking segments and various loop types in the hybrid-type human telomeric G-quadruplexes for binding specificity is highlighted. On the other hand, both EPI and Pt-tripod recruit a DNA base to form a ligand-base plane covering the external G-tetrad. Both the EPI and the Pt-tripod scaffolds cover only two guanine bases and by recruiting an adenine residue, a full tetrad coverage is achieved. Such a phenomenon has also been observed for ligand complexes of the parallel-stranded c-MYC promoter G-quadruplex [71,72]. The engagement of the target-DNA residue anchors the specific orientation of the ligand and might be the basis for the specific recognition of a ligand. In contrast to quadruplex ligands with large aromatic systems covering most of the tetrad, the asymmetric crescent shaped EPI or the tri-armed Pt-tripod facilitate this target-base recruitment.

**Figure 8:**
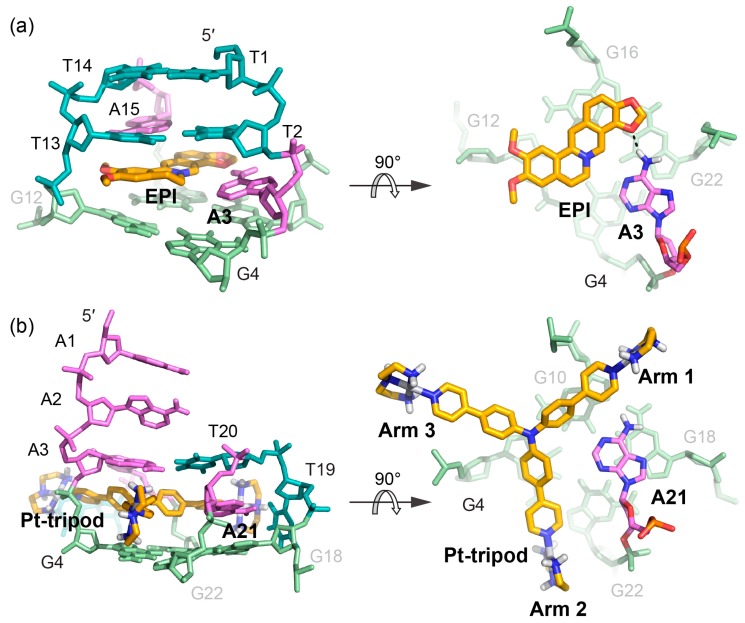
Comparison of the 5′-binding modes between EPI and Pt-tripod with human telomeric hybrid-type G-quadruplexes (**a**) Upon binding, the 5′-flanking A3 is recruited by EPI and forms a quasi-triad plane (right). The multi-layer drug-binding pocket for EPI is formed by the 5′-end flanking residues and TTA lateral loop. (left). Potential hydrogen bond is shown as the black dashed line. (**b**) Upon binding, A21 in the second lateral loop is recruited by Pt-tripod and forms a quasi-triad plane (right), stacking on top of the 5′-external G-tetrad and locking the position of Pt-tripod. Each arm of Pt-tripod stretches into different grooves. The Pt-tripod:A21 plane is capped by the A3:A9:T20 triad (left).

Comparing how EPI and Pt-tripod bind in the 5′-complexes of the two hybrid-type human telomeric quadruplexes reveals similarities and critical differences in stacking, hydrogen bond, and electrostatic interactions (Figure 8). Similarly, both drugs stack upon two guanine bases of the 5′-tetrad and recruit an adenine base to form a ligand-base plane covering the 5′-tetrad. However, their binding positions and interactions are different. The more compact and planar EPI intercalates between the second lateral loop and the 5′-tetrad and recruits A3 from the 5′-flanking segment in the hybrid-2 structure. EPI forms a clear hydrogen bond with A3 in the ligand-base plane, while the T13 and A15 of the second lateral loop provide an optimal capping covering the long axis of EPI. In contrast, the tri-armed Pt-tripod intercalates within the 5′-flanking segment between A3 and the 5′-tetrad, and recruits A21 from the lateral loop in the hybrid-1 structure using two arms in a more shape-complementary manner. Thereby, the EPI molecule is opposite to the 5′-flanking strand anchoring towards the A3, while the Pt-tripod is opposite to and restricted by the lateral loop above the 5′ external tetrad. Both ligand-base planes are covered by a triad of flanking and loop residues, each of which including a reverse Watson–Crick AT base pair. Stacking interactions are more predominant for the planar EPI in its interactions with the 5′-tetrad and the capping triad than for the non-planar Pt-tripod (Figure 4c). In the EPI complex, the capping triad is more stable and well-defined, as the three bases are all connected through hydrogen-bonds, and is covered by an additional layer of T:T base pair. This extensive binding pocket defines the stable complex formation of the EPI and hybrid-2 structure, which promotes the EPI-induced conversion of other human telomeric quadruplex topologies towards the hybrid-2 folding. In contrast, in the capping triad of the Pt-tripod 5′-complex, the A3:T20 base pair is distant from the A9 residue, which likely stabilizes the arm 3 by stacking with its aromatic moiety (Figure 6b).

The positive charge of the two drugs contributes to their respective binding interactions in different ways. The positively charged nitrogen of EPI is located above the central pore close to the negatively polarized carbonyl functional groups of the 5′-tetrad, analogous to the centrally coordinated cations. For Pt-tripod, the central moiety is uncharged and also moved to the tetrad edge. Instead, its long arms position the three terminal positively charged Pt(II) complexes for interactions with negatively charged phosphate backbones of three different G-quadruplex grooves. Although cationic drugs might have general binding to other nucleic acids structures, this groove-anchoring mode may be used to enable further G-quadruplex selectivity. For example, cationic side chain might be added in an EPI derivative to similarly anchor it to the grooves of the hybrid-2 quadruplex.

In summary, the reviewed high-resolution complex structures of the hybrid-type human telomeric G-quadruplexes with the EPI and the Pt-tripod small molecules advance our knowledge about quadruplex-ligand interactions. Both drugs induce a previously unprecedented rearrangement of the capping residues to form extensive multi-layer binding pockets, with each drug recruiting a G-quadruplex DNA residue to form a ligand-base plane and define its respective binding position. Additional features observed for each drug are the EPI-induced conversion of alternative human telomeric quadruplex topologies towards the hybrid-2 type and the Pt-tripod-induced 3′–3′ dimerization to form a 4:2 complex. The identified mechanisms of molecular recognition will provide insights into designing improved cancer therapeutics targeting the human telomeric G-quadruplexes.
